# Humeral metastases: an unusual presentation of pancreatic cancer

**DOI:** 10.1093/jscr/rjac157

**Published:** 2022-05-25

**Authors:** Jonathan A McCafferty, Matthew D Free, Daniel J De Villiers, Prue P A Keith

**Affiliations:** Department of Orthopaedics, Northeast Health Wangaratta, Wangaratta, Victoria, Australia; Department of Orthopaedics, Northeast Health Wangaratta, Wangaratta, Victoria, Australia; Department of Orthopaedics, Northeast Health Wangaratta, Wangaratta, Victoria, Australia; Department of Orthopaedics, Northeast Health Wangaratta, Wangaratta, Victoria, Australia

## Abstract

In patients presenting with symptomatic bone metastases, the usual primary malignancies are breast, prostate, thyroid, lung and kidney. Pancreatic cancers are an uncommon cause of bone metastasis and, when they are, it is typically to the axial skeleton. We present the case of a 77-year-old woman who presented to our emergency department with right arm pain. Investigation demonstrated a metastatic lesion with impending pathological fracture. Further investigation found a necrotic pancreatic mass in the tail of the pancreas. Histopathology from the humeral metastasis was consistent with metastatic adenosquamous carcinoma of pancreatic origin. The humerus metastasis was successfully managed with an intramedullary nail. This case highlights an unusual presentation of pancreatic adenocarcinoma with humeral bone metastasis.

## INTRODUCTION

The incidence of pancreatic cancer is increasing in Australia. Pancreatic ductal adenocarcinoma is the most common type of pancreatic cancer. The most common malignancies associated with skeletal metastases are breast, prostate, thyroid, lung and kidney. Pancreatic cancer is less often associated with skeletal metastases, however up to 20% of pancreatic cancer are thought to have bone metastases. We present a case of pancreatic adenocarcinoma presenting with a symptomatic humeral metastasis.

## CASE REPORT

A 77-year-old woman was admitted to our Regional Centre under a multi-disciplinary team, for investigation of functional decline, associated with symptomatically severe right arm pain and impaired liver function tests (LFTs). On specific questioning she reported generalized malaise, loss of appetite and weight loss over the prior 2 weeks. There was no other relevant past history in respect to medical or social factors. Prior to this admission she lived entirely independently.

Examination revealed proximal humeral tenderness and reduced active shoulder and elbow range of motion secondary to pain inhibition. There were no neurological deficits.

Index haematology demonstrated a normal full blood examination and renal function. The LFTs were abnormal and demonstrated a cholestatic picture (Bilirubin, BR 12 μmol/l, ALT 53 U/l, AST 63 U/l, ALP 496 U/l and GGT 435 U/l). The Tumour markers were elevated with cancer antigen (CA) 19–92 590 U/ml (normal <38 U/ml), CA-1252420 (0–35 U/ml) and carcino embryonic antigen (CEA) 385 ug/l (<10 μg/l).

**Figure 1:**
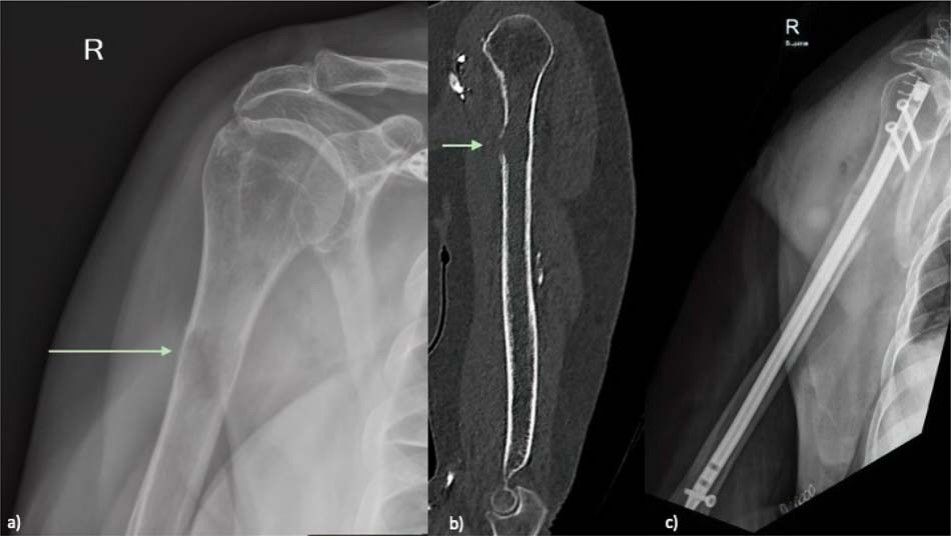
(**a**) Plain radiograph of the right humerus demonstrating a lucent lesion in the proximal humerus with cortical destruction indicated by green arrow (**b**) Sagittal CT of the right humerus showing a proximal humeral lesion with breach of the anterior cortex (green arrow) at high risk of pathological fracture (**c**) plain radiograph Day 1 post prophylactic intramedullary nail of right humerus.

**Figure 2:**
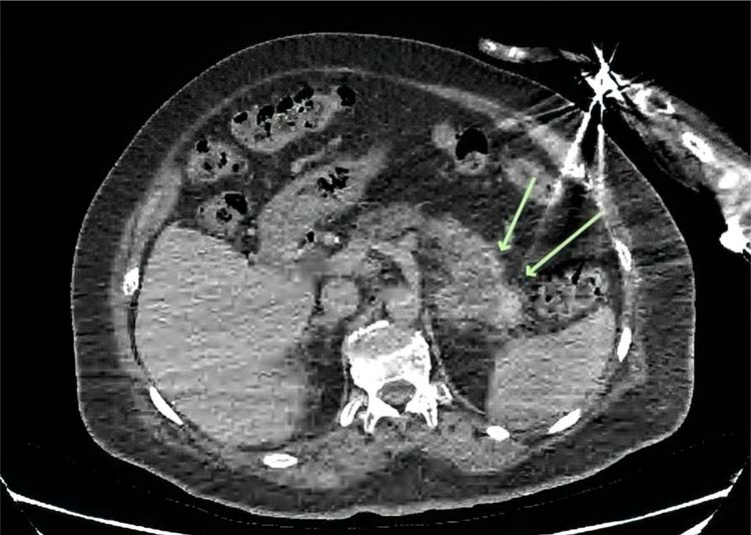
Axial CT of the abdomen demonstrating a necrotic mass centred in the tail of the pancreas measuring 57 × 43 mm (green arrows).

An X-ray of the right humerus demonstrated a lytic lesion in the proximal humeral diaphysis with metastatic features (Fig. 1a). Subsequent computed tomography (CT) of the right humerus demonstrated a medullary metastasis at high risk of pathological fracture (Fig. 1b). Further imaging with CT chest–abdomen–pelvis demonstrated a necrotic mass in the tail of the pancreas (Fig. 2) with direct extension into the left adrenal gland and suspected liver metastases.

Due to the calculated fracture risk, a prophylactic intramedullary humeral nail was recommended and performed [1]. Tissue samples were obtained by focal reaming and curettage and sent for histopathology. A diagnosis of metastatic adenosquamous carcinoma with mucoepidermoid morphology of pancreatic origin was made.

The postoperative recovery was uneventful with significant improvement of function in the right upper limb.

Conservative and symptomatic management was decided upon at the Medical Oncology Multidisciplinary Meeting.

## DISCUSSION

The incidence of pancreatic cancer is increasing worldwide. In Australia, pancreatic cancer is the eighth most common cancer in both men and women, and the fifth most common cause of cancer death [2]. Pancreatic ductal adenocarcinomas account for most pancreatic malignancies, with the adenosquamous carcinoma (ASC) subtype comprising 0.4–4% of cases [3]. ASC is defined histologically as containing 30% or more squamous cell carcinoma and adenocarcinoma [3]. It occurs equally in men and women, with an average age of 66 years at presentation [4]. ASC most commonly occur in the head of the pancreas, however compared to adenocarcinoma it is more likely to be in the body/tail of the pancreas. More than 50% of ductal adenocarcinoma and ASC have metastasized at initial presentation [4]. Both ASC and adenocarcinoma have improved survival following surgical resection, but ASC has poorer long-term survival [4].

In the setting of metastatic bone disease, pancreatic cancer is less likely to be the primary malignancy. The most common tumours associated with bone metastases are breast, prostate, thyroid, lung and kidney [5]. Of this group, breast and prostate cancer are thought to account for > 80% [5]. Ryan *et al*. performed a large retrospective analysis of patients with bone metastases present at the time of cancer diagnosis [6]. In this setting, the most common primary malignancies with bone metastases were lung cancer (8.7 per 100 000) followed by prostate and breast cancer (3.19 and 2.38 per 100 000) [6].

Pancreatic metastatic bone disease is underappreciated. In an autopsy study in patients with pancreatic cancer, Blastik *et al*. demonstrated 83.8% of cases with pancreatic cancer had metastasized, with bone metastases in 4.5% of patients [7]. Some studies report that between 5 and 20% of patients with pancreatic cancer will have skeletal metastases, with a primary in the tail more likely to metastasize to the bone [8, 9].

The majority of patients with bone metastases from pancreatic cancer are symptomatic and are associated with at least one other site of metastases [9]. A single centre cohort study of patients with bone metastases from pancreatic cancer demonstrated that most metastases were to the axial skeleton, with only 11% of patients having appendicular metastases [10]. Borad *et al*. demonstrated a similar pattern of bone metastases with the spine, pelvis and ribs most affected and upper limb metastasis seen in only 14% of patients with pancreatic bone metastases [9]. Bone metastasis is associated with worse survival in patients with pancreatic cancer, with a median survival of 7 months [10].

This case highlights an unusual presentation of metastatic pancreatic cancer with a symptomatic humeral metastasis and impending fracture. Bone metastases at the time of diagnosis and involving the appendicular skeleton are uncommon in pancreatic cancer. We present this case to improve awareness around this tumour.
